# A clinical assessment of cochlear implant recipient performance: implications for individualized map settings in specific environments

**DOI:** 10.1007/s00405-016-4130-2

**Published:** 2016-06-08

**Authors:** Matthias Hey, Thomas Hocke, Stefan Mauger, Joachim Müller-Deile

**Affiliations:** 1 Department of Otorhinolaryngology, Head and Neck Surgery, Audiology, Christian-Albrechts-University, Kiel, Germany; 2 Cochlear Deutschland, Hannover, Germany; 3 Cochlear Limited, Melbourne, Australia; 4 Audiology Consultant, Kiel, Germany

**Keywords:** Cochlear implant, Speech intelligibility, Individual programming, Signal processing, Speech, Audiometry

## Abstract

Individual speech intelligibility was measured in quiet and noise for cochlear Implant recipients upgrading from the Freedom to the CP900 series sound processor. The postlingually deafened participants (*n* = 23) used either Nucleus CI24RE or CI512 cochlear implant, and currently wore a Freedom sound processor. A significant group mean improvement in speech intelligibility was found in quiet (Freiburg monosyllabic words at 50 dB_SPL_) and in noise (adaptive Oldenburger sentences in noise) for the two CP900 series SmartSound programs compared to the Freedom program. Further analysis was carried out on individual’s speech intelligibility outcomes in quiet and in noise. Results showed a significant improvement or decrement for some recipients when upgrading to the new programs. To further increase speech intelligibility outcomes when upgrading, an enhanced upgrade procedure is proposed that includes additional testing with different signal-processing schemes. Implications of this research are that future automated scene analysis and switching technologies could provide additional performance improvements by introducing individualized scene-dependent settings.

## Introduction

### Cochlear implant performance

Cochlear implantation is currently a well-established method for restoring hearing to people with profound hearing loss. Substantial open-set speech understanding in quiet is achieved by the majority of cochlear implant (CI) recipients [1, 2]. Speech perception in noisy environments is still a challenge, since it degrades more quickly for CI users than with normal listeners [3]. Some performance improvements in noise can be achieved with current CI systems through the use of sound processing technologies, such as dynamic range optimization [4–6], noise reduction [7–9], and multi-microphone beam-forming techniques [10, 11]. Connected to this technological progress is an additional programming and speech-testing effort for individual recipients: The application of signal processing does not lead to an improvement in every individual case [11]. In other words, certain CI recipients may benefit from settings different to the mean findings. The required extensive speech testing leads to some methodological issues reasoned, as there are repeated testing with limited speech material [12–14]. With that said, the integration of CI fitting in clinical routine is a topic with changing boundary conditions.

### Environment-specific programs

While providing benefit to CI recipients, the growing number of front-end sound processing technologies requires additional effort. Audiologists need to explain to the recipient which sound processor program is best suited for a certain listening situation. CI recipients then need to understand the listening environment they are in, and then to decide which is the most appropriate program. A recently introduced solution is a scene classifier technology which detects the listening environment, and automatically selects a suitable program [15]. Significant improvements in performance and acceptance have been found through the automatic selection of directional microphones, wind noise reduction, and newly introduced technologies, such as noise reduction, which is enabled in all scenes. As previously shown, current signal-processing algorithms provide a mean benefit for a group of recipients. However, not each and every recipient’s outcome corresponds to the mean findings [5, 6, 11, 16].

### Individual performance outcomes

Noise reduction has shown significant improvements in group performance typically between 1 and 2 dB_SNR_ [7, 15], and has also shown improvements in listening quality [8, 17]. Although group results have been strong, there has always been a range of outcomes in quiet or in noise, with some recipients receiving significant individual speech intelligibility improvements or decrements when upgrading to a program with this technology [11, 15].

Similarly, previous studies have shown individual preference or performance differences for front-end processing technologies [5, 16] and significant individual performance differences with dynamic range settings [18].

### Hypothesis and testing

Recently, a new noise-reduction algorithm (signal-to-noise-ratio noise-reduction: SNR-NR) was introduced to the SmartSound iQ suite. As already shown, this algorithm [11], as well as other algorithms [6, 19], result in a range of individual outcomes. In addition to previous group analysis studies, this study investigates noise reduction with focus on individual benefit. Therefore, the aim of this study is to determine if an individual’s performance outcome with a range of signal-processing schemes is different to outcomes with the default technology selection. The study was designed to answer the following questions:

- Whether individuals are found to perform better with or without noise reduction for speech in quiet and in noise.
- The proportion of individuals with scene-specific improvements from noise reduction.
- The additional testing effort needed to achieve individually optimized scene classification settings.

This study was performed at the time of upgrading, so individual performance between the previous Freedom processor and the new CP900 series processor could be investigated.

## Methods

### Participants

The CI recipient group consists of 23 research participants from the clinical patient pool. All recipients were scheduled for a regular upgrade procedure between July 2013 and August 2014. The data were analyzed retrospectively with local ethics approval (D 488/14). All procedures performed in studies involving human participants were in accordance with the ethical standards of the institutional and/or national research committee and with the 1964 Helsinki declaration and its later amendments or comparable ethical standards.

Selection criteria for this study were postlingual onset of deafness, implantation with a Nucleus CI24RE, or CI512 cochlear implant (Cochlear Ltd., Australia), and being a current user of a Freedom sound processor. In addition, patients had to demonstrate sentence intelligibility scores of 75 % with the Freedom sound processor or greater in Oldenburger sentence test (Olsa) in quiet at 65 dB_SPL_. Bilateral implantation was not an exclusion criterion. All patients had a full insertion of their electrode array, and all electrodes were switched on except for patient #1 who had one electrode switched off).

Preoperatively, all subjects had demonstrated a speech intelligibility level, as measured by the Freiburg Monosyllable Test [20], of 40 % or less in both ears at 65 dB_SPL_ with their optimally adjusted hearing aids. Participants mean age was 54 ± 23 years, 11 patients were unilaterally implanted, and 12 were bilaterally implanted. All participants had at least 4 years’ experience with their CI, with a group mean of seven years experience. All but one (#13, MP3000) were using the ACE coding strategy with individually fitted stimulation rate and number of maxima selected through user preference. Table 1 summarizes biographical details for research participants in this study.

**Table 1:** Recipients biographical data

Patient	Age (years)	Usage of CI (years)	Side	Gender	Rate (pps)	Maxima
#1	71.0	8.0	Right	m	1800	8
#2	43.2	8.9	Left	f	1200	12
#3	84.4	6.3	Right	m	1200	12
#4	64.9	5.8	Right	m	1200	12
#5	64.9	6.1	Left	m	1200	12
#6	73.0	4.9	Right	f	1200	12
#7	76.9	5.7	Left	f	1200	12
#8	11.7	4.8	Left	f	900	10
#9	13.9	8.2	Left	f	1200	12
#10	75.9	4.6	Left	m	1200	12
#11	60.0	8.2	Right	f	1200	12
#12	60.0	7.0	Left	f	1200	12
#13	62.0	7.2	Left	m	1200	7
#14	52.8	6.5	Right	m	1200	12
#15	82.4	6.2	Left	m	1200	12
#16	44.9	7.9	Left	f	1200	12
#17	36.3	8.4	Left	f	500	12
#18	61.3	6.1	Left	m	1200	12
#19	17.7	7.7	Right	m	900	8
#20	30.5	6.3	Left	f	1200	12
#21	61.6	7.3	Left	f	1200	12
#22	70.5	7.1	Right	f	1200	12
#23	16.9	6.0	Left	m	1200	12

### Test procedures

The study used a single-subject, repeated measures design with subjects serving as their own control. Testing was conducted across three test sessions spaced between 2 and 3 weeks.

All tests in quiet and in noise were conducted in a soundproof test booth via calibrated loud speakers. Freiburg words in quiet (DIN 45621-1 and 45626-1) were presented through a computer-based implementation of the Freiburg words test (Equinox audiometer; Interacoustics, Denmark and evidENT 3 software, Merz Medizintechnik, Germany). Each word list consists of 20 words. One list of words was presented for each of the fixed levels of 40, 50, 60, 70, and 80 dB_SPL_ in the free-field condition with the loudspeaker placed 1 m in front of the recipients. For each list, the percent correct score was determined. Research participants implanted on both ears were fitted bilaterally with the new sound processors and used the two new sound processors for take-home adaptation, but they were tested unilaterally on each side with the contralateral sound processor being switched off.

Sentences in noise were presented using the “Oldenburger Messprogramme” software (Hörtech, Oldenburg, Germany) to control the recording and to calculate the speech reception threshold (SRT). The Olsa was performed in background noise using lists of 30 sentences. The Oldenburg speech-spectrum shaped noise was fixed at a constant presentation level of 65 dB_SPL_. Both speech and noise signal were presented from the front. The SRT was measured using an adaptive procedure [21], starting at a signal-to-noise ratio (SNR) of 0 dB_SNR_. The level of the speech signal was decreased when more than two of the five words were recognized correctly; otherwise, it was increased according to the test guidelines [21]. The SRT was defined as the SNR resulting in a 50 % words correct score. All subjects were accustomed to the test procedure, having been previously assessed three or more times as part of our clinical routine, (both in quiet and in noise, listening to at least 80 sentences each time) in alignment with the training practice. To further ensure sufficient familiarity with the Olsa  [14], a training session was performed immediately before testing in noise, by administering 30 recorded sentences in quiet at a presentation level of 65 dB_SPL_ in every test session.

Three programs were tested and compared in this study (Table 2). All participants were current users of ADRO in their Freedom processor. The first program used the ‘Standard’ directional microphone setting and ADRO, and will be referred to as ‘Freedom ADRO’. The CP900 series processors were programmed using CustomSound (Cochlear Ltd., Australia). Two CP900 series (CP9) custom programs were tested. One custom program did not use the default signal processing as proposed by the Custom Sound Software. Instead it used Standard directionality and ADRO, and will be referred to as ‘CP9 ADRO’ (Table 2). The other CP900 series custom program used Standard directionality, ADRO, and SNR-NR, and will be referred to as ‘CP9 NR’ (Table 2). This program also used a modified T-SPL (threshold sound pressure level) of 15 dB in contrast to the default T-SPL of 25 dB. The T-SPL modification was selected as result of a pilot study. With the default T-SPL at 25 dB, the noise reduction reduced signals below threshold, so that some participants reported the sounds being too soft in low noise environments, and speech intelligibility was degraded for low levels in quiet by about 10 % points for some recipients. The lower T-SPL re-introduced low-level environmental noise to participants and increased speech recognition results at 40 and 50 dB_SPL_.

**Table 2:** Sound processor settings for testing speech perception in quiet and noise introducing different SmartSound options

Processor condition	SmartSound technologies		
Freedom ADRO	ADRO	T-SPL 25 dB	
CP9 ADRO	ADRO	T-SPL 25 dB	
CP9 NR	ADRO	T-SPL 15 dB	SNR-NR

Prior to the first test session, the Freedom processors were tested to ensure correct operation. If a fault was identified, the given component was replaced. At the first test session, participants were tested with words in quiet and sentences in noise with their Freedom processor. This condition served as a baseline control. They were then upgraded from the Freedom to the Nucleus 6 sound processor. Program selection was randomized between CP9 ADRO and CP9 NR. Participants then used the new program in their daily life for at least 2 weeks to acclimatize to the new signal-processing technologies. Participants were then tested again at a second test session with their CP9 program with words in quiet and sentences in noise. They were then programed with the remaining CP9 program, and recalled for a final test session where they were tested again with words in quiet and sentences in noise after a further two weeks acclimatization. Additional to participants test program, they also received a second program (P2), which had the same smart sound option as the test program with the addition of the automated scene classification technology (SCAN) to become familiar with the advanced features of the CP9.

### Data analysis

To determine the critical difference for individual speech comprehension outcomes, the test–retest accuracy for both speech tests was needed. This was found by calculating the standard deviation of the differences between test and retest pairs (multiplied by 1.96) resulting in the two-sided critical difference at the 95 % confidence level [11]. For words in quiet, a critical difference of 24 % was used based on a standard deviation for test–retest difference of 12 % (Schmidt 2015—unpublished data). A critical difference of 2.2 dB was used for the Oldenburger test in noise [14].

To determine group performance outcomes, a repeated measures ANOVA was utilized. Global test for comparison of all three hearing programs with the test version of Greenhouse-Geisser and post-hoc pair-wise comparisons based on estimated marginal means was introduced. The (unadjusted) pair-wise comparisons have only been considered if the global test was significant. In the special case of three groups, this procedure ensures the control of the family wise type I error rate over all pair-wise comparisons following arguments of the closed testing principle [22]. An alpha value of 0.05 was used to determine significance. All comparisons used a two-tailed analysis.

## Results

### Speech understanding in quiet

**Fig. 1:**
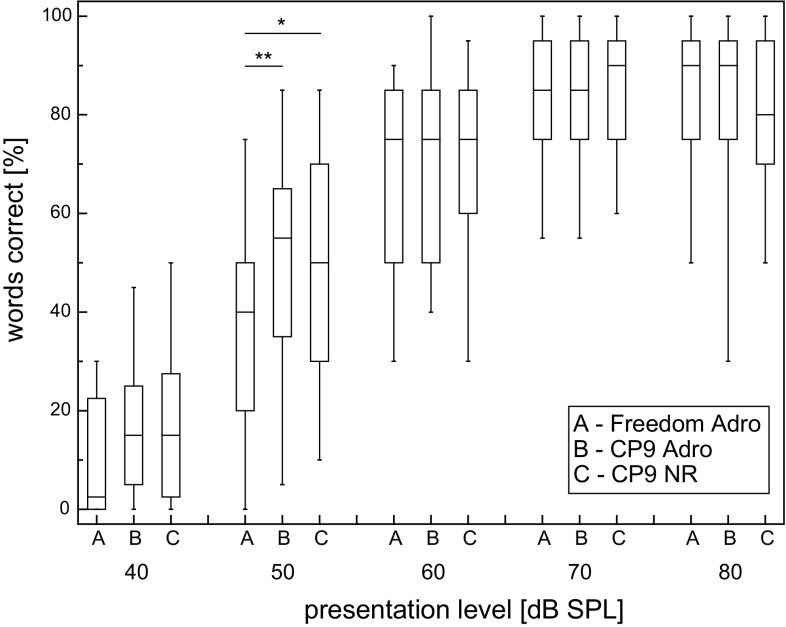
Speech intelligibility of Freiburg monosyllabic words in the free-field condition depending on presentation level for the Freedom Adro program and two CP9 programs using different SmartSound options. *Boxplots* show median, 1st and 3rd quartile, minimum, and maximum (*N* = 23)

Group mean outcomes with the three programs tested for words in quiet at 40–80 dB_SPL_ in steps of 10 dB_SPL_ are presented in Fig. 1. The mean discrimination functions show a monotonic increase in speech understanding up to 70 dB_SPL_ for all three processor settings, followed by a plateau between 70 and 80 dB_SPL_. Analysis of the speech recognition rate at 40 dB_SPL_ showed a floor effect. Ceiling effects were found for presentation levels of 60 dB_SPL_ and above. This is demonstrated by the skewness of the boxplots in Fig. 1.

The data for each presentation level were checked for their distribution of measurements. The boxplots of the 50 dB_SPL_ showed a strong similarity to the normal distribution; therefore, ANOVA models are applicable for this presentation level. Kolmogorov–Smirnov test detected no significance in any of the variables (*p* < 0.01). The data for the monosyllabic words at 50 dB_SPL_ were taken for further analysis, as they were unaffected by floor and ceiling effects.

The Greenhouse-Geisser test of within-subjects effects (Fig. 1) was significant (*p* = 0.015, *F* = 4.83, *df* = 1.84). Analysis gives a statistical significant improvement of 13 % at 50 dB_SPL_ for the CP9 ADRO (*p* = 0.003) and of 10 % for CP9 NR (*p* = 0.04) condition in comparison to Freedom ADRO. The comparison of CP9 ADRO and CP9 NR at 50 dB_SPL_ showed no statistical significant difference (*p* = 0.58) in speech comprehension in quiet.

### Speech understanding in noise

**Fig. 2:**
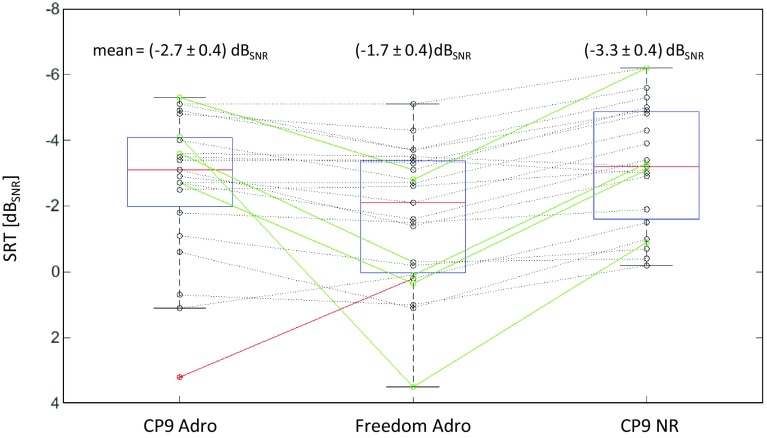
SRT scores for the Olsa in noise using different processor settings. Test signals were presented in free field with speech and noise coming from front. Boxplots show median with 1st and 3rd quartiles, minimum and maximum. Means of SRT and standard deviation of the mean are shown at the *top* of each program. Individual results are connected via line (*green line* significant improvement of SRT; *black dotted line* no significant change; *red line* significant deterioration of SRT). *N* = 23

Mean SRT for Freedom ADRO was (−1.7 ± 0.4) dB_SNR_. The Greenhouse-Geisser test of within-subjects effects (Fig. 2) was significant (*p* = 0.001, F = 10.2, *df* = 1.62). The mean SRT improvement was 1.0 dB_SNR_ for CP9 ADRO (*p* = 0.02) and 1.6 dB_SNR_ for CP9 NR (*p* < 0.001). No statistical significant difference in SRT was found between the two Nucleus 6 SmartSound options (*p* = 0.16) (Fig. 2).

### Relationship between speech understanding in quiet and in noise

**Fig. 3:**
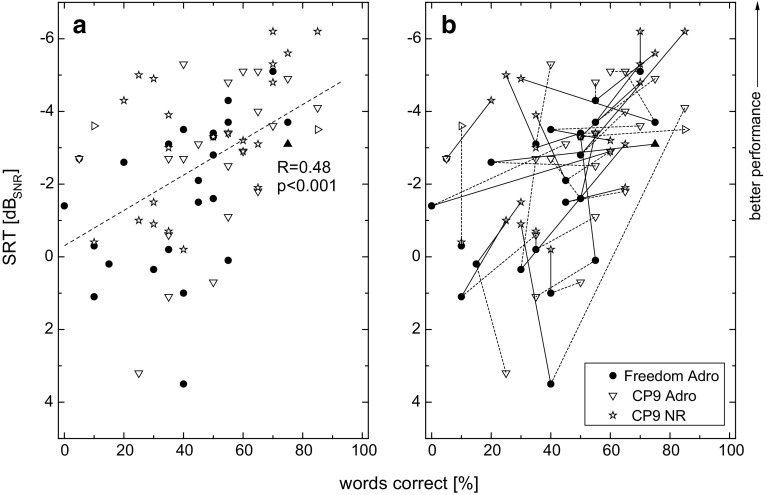
Speech intelligibility in noise plotted against speech intelligibility in quiet for the three programs tested. SRT scores with Olsa sentences in 65 dB_SPL_ noise and results of Freiburg monosyllabic word score at 50 dB_SPL_ are presented. **a** Linear regression line for all data points plotted as *dotted line* (its correlation coefficient and significance level are shown). **b** Individual results are connected showing for each baseline result with the Freedom processor two resulting data points with the CP9 ADRO (*dotted line*) and for CP9 NR (*straight line*) programs

Individual results for speech in quiet and speech in noise are plotted for each of the three processor settings in Fig. 3a. A significant correlation between speech understanding in quiet and in noise was found, with a correlation coefficient of 0.48. Although this result is significant (*p* < 0.0001), a large degree of variability in the data is evident showing no strict connection between the two measures.

In Fig. 3b, the same data are plotted, with the addition of connecting lines between the three programs tested for each individual. This figure shows that although a significant correlation was found across all subjects and all programs (Fig. 3a), and across programs for an individual, a wide range of possible performance changes were found.

### Individual intelligibility and its optimization in quiet and noise

**Fig. 4:**
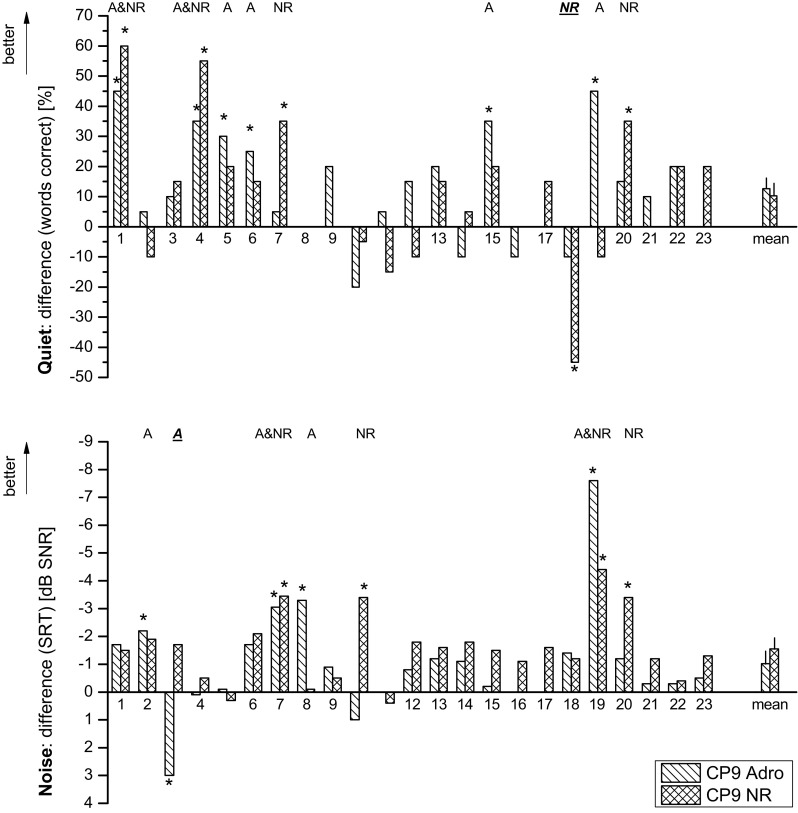
Difference between speech comprehensions compared to the Freedom processor for CP9 Adro (**a**) and CP9 NR (NR) program in quiet (*top)* and noise (*bottom*) for 23 recipients. Significant improvements compared to the Freedom ADRO program are marked with *normal text* and decrements are marked with *cursive* and *underlined text* above individual results

As patients show different baseline understandings and different results after conversion to CP9 programs, it is useful to analyze individual benefits. To assess the upgrade benefit, the differences of CP9 understanding relative to individuals Freedom ADRO performance in quiet and noise are shown in Fig. 4.

To determine if an individual had a significant improvement or decrement in performance, the critical difference for the word and sentence tests has to be exceeded. This removes test variability and assesses the individual performance. An upgrade would be considered successful if the speech understanding in noise and quiet improved, or if the speech understanding improved in one situation without decrement in the other for at least one program.

In quiet using CP9 ADRO, six of 23 CI patients showed an improvement and for CP9 NR, four out of 23 patients improved with the CP9. For the CP9 NR option, one out of 23 patients showed a decrement in the test result in quiet.

In noise, four of 23 patients showed an improvement for both the CP9 ADRO and the CP9 NR condition. One out of the 23 patients tested showed a decrement in the CP9 ADRO condition. These improvements were not consistent for both CP9 programs, meaning an improvement using the CP9 ADRO is not necessarily connected with an improvement in the CP9 NR program, or vice versa. 11 of the 23 patients showed an individual improvement in quiet or in noise with in at least one of the programs tested: CP9 ADRO and CP9 NR.

On the other hand, participant #3 showed a decrement for CP9 ADRO in noise, but a non-significant mean improvement for CP9 NR in quiet and in noise. A comparable but inverse situation found a significant decrement of patient #18 in quiet in the CP9 NR condition. This patient showed a non-significant mean improvement in noise and non-significant mean decrement in quiet for CP9 ADRO.

To summarize the individual data in Fig. 4, the number and percentages of individuals with significant changes are shown in Table 3 for the CP9 ADRO and CP9 NR programs.

**Table 3:** Grouped results after conversion from freedom to CP9

	CP9 Adro (subject count %)	CP9 NR (subject count %)
Sign. improvement (quiet)	6/26	4/17
Sign. improvement (noise)	4/17	4/17
Sign. decrement (quiet)	0/0	1/4
Sign. decrement (noise)	1/4	0/0
Sign. improvement (quiet or noise)	9/39	6/26
Sign. decrement (quiet or noise)	1/4	1/4

By fitting all recipients with CP9 ADRO, 39 % were found to receive a significant individual improvement in quiet or in noise and 4 % were found to receive a decrement in noise. By fitting CP9 NR to all recipients, 26 % were found to receive a significant individual improvement in quiet or in noise and 4 %were found to have a decrement in quiet. No one was found to show a decrement in quiet and in noise using CP9 ADRO as well as CP9 NR.

Six out of the 23 patients showed a greater improvement using CP9 ADRO than using the default processor setting of the CoustomSound programming system (CP9 NR). This is an individualized improvement in intelligibility in quiet and in noise by fitting just one program, but it requires testing in both listening situations.

## Discussion

Overall, the sound processor conversion from Freedom processor to CP9 processor was found to be a beneficial procedure for all patients. New signal-processing algorithms were found to provide mean improvements of speech understanding in quiet and noise as reported by previous studies [14]. Nonetheless, there are some additional details that should be paid attention, and will be discussed hereafter.

Speech understanding in quiet should be, additionally, tested at levels below 65 dB changed patient selection criteria, and considerable preoperative speech recognition scores [23, 24] all contribute to ceiling effects  [25] of speech tests in quiet at 65 dB. At levels from 70 to 80 dB_SPL_, we see saturation at a mean score above 80 %. Beside methodological aspects, the relevance of lower levels in recipient’s everyday life [26] should also be considered. Figure 1 shows a floor effect for 40 dB_SPL_ and a ceiling effect at 60 dB_SPL_ presentation level already. In this study, the largest difference between programs was found at a presentation level of 50 dB_SPL_. For these reasons, this speech presentation level was considered the most suitable for analysis, while still being representative of a real-world environment. Our results complement recent findings with the same technologies [15], as they show an improvement at lower presentation levels in quiet while maintaining an overall benefit in noise, Fig. 2. At a first glance, this finding suggests a straightforward procedure for upgrading CI recipients with new processor technologies.

However, Fig. 3 reveals an additional aspect for clinical routine processor upgrades. Because of the significant but weak correlation between speech understanding in quiet and speech understanding in noise in Fig. 3a, outcomes should be obtained for both hearing situations. Furthermore, Fig. 3b shows that there is no strict predictable individual performance outcome after the upgrade procedure from Freedom to CP9.

Table 3 gives an overview for an improvement with one default setting for all recipients. If, for example, CP9 ADRO is chosen as the default setting, about 39 % of the recipients would have shown a significant improvement on an individual basis in quiet or noise. If CP9 NR is chosen as the default setting, about 26 % of the recipients would have shown a significant improvement on an individual basis in quiet or noise. These numbers are comparable to other findings [6, 11], where up to 30 % of the recipients showed an individual significant benefit in a certain hearing situation.

At this very point, a clinic may decide for one of the processor settings for all recipients involved in an upgrade procedure. Therefore, no additional testing might be required, as we assume a mean improvement for all recipients, as shown in Figs. 1 and 2.

In a number of countries, such as in Germany, clinics have to demonstrate an individual significant benefit for recipients to receive funding from their health insurance provider for upgrades. A minimum test process with the previous and the new processor in quiet and noise is typically required, resulting in four test conditions. This procedure would result in approximately 25–40 % successful upgrades at a 95 % significance level with collocated speech and noise testing described in this study. In testing two programs of the new sound processor, one has to test six conditions. This would result in a significant improvement for nearly 50 % (11/23) of the recipients, when using either a CP9 Adro or CP9 NR program (see Fig. 4). An increment of 33 % of testing effort results in approximately 25 % increment of numbers of successful upgraded recipients when testing both programs in contrast to CP9 NR only.

Another consideration is improvements due to individual program selection. This could be done for either scenes, or for different programs in each scene. To test this, two programs were investigated in two acoustic scenes.

When selecting the individual optimized program, no patient showed a significant decrement in performance when using the best selected program and eleven of the tested 23 patients showed a significant improvement in at least one condition.

However, it was also shown that best speech understanding in quiet is not provided by CP9 ADRO in all cases, even if ADRO [5, 6] was originally intended to improve speech understanding in quiet. Two of the recipients, #7 and #20, showed an exclusive improvement in quiet using the CP9 NR option. The same aspect was found in noise. Two of the recipients, #2 and #8, showed an exclusive improvement in noise using CP9 ADRO instead of also with the designated noise-reduction algorithm [11] in the CP9 NR program. Those four recipients are remarkable, since they all showed a deviating behavior in the preferred preprocessing scheme.

A more important scenario than a missing significant improvement is the significant performance decrement. This was found in two recipients: Recipient #3 with a significant decrement of speech understanding in noise using CP9 ADRO shown in Fig. 4. This patient should be then fitted with CP9 NR. Patient #18 showed a significant decrement in quiet using CP9 NR. Therefore, this recipient should use CP9 ADRO.

In some of the significant cases, one of the two processing schemes is better in both tested listening situations, quiet and noise.

An important conclusion for the clinical upgrade procedure can be drawn at this stage.

Additional speech testing conditions are not only about the question of showing significant individual improvements during upgrade and testing new signal-processing algorithms. They are also about preventing significant decrements of speech scores in single recipients. With an extended audiometric testing procedure, it can be seen that significant performance improvements could be achieved for individuals using the CP9 processor.

Automatic scene classification default technology selection does show significant performance improvements [15], and, therefore, are suitable as defaults. However, due to significant individual preference and performance variability, additional gains are expected to be possible for some recipients. This could be achieved through an automatic scene classification technology which selects the optimal technologies for a recipient in each listening scene. For instance, if an individual was found to perform better in noise, but worse in quiet with noise reduction enabled, an automatic scene classifier could deactivate noise reduction in the quiet scene. Similarly, if an individual was found to perform better in quiet, but worse in noise with noise reduction, the automatic scene classifier could deactivate noise reduction in noise. This method could provide individual specificity to optimize the automatic system to provide additional benefits for individuals.

To achieve this, performance and/or preference outcomes would need to be found in listening environments indicative of each scene class: Speech, Speech in noise, Noise, Quiet, Wind, and Music. Such a method would be accurate, but would also take longer than typically available for program fitting. Finding tests which predict broad preference or performance outcomes would be important to make such a method clinically feasible. For instance, a test could be performed in one scene class, and program selection could be predicted with high accuracy due to known correlations for other scene classes. Alternatively, information from objective measure tests may prove to be correlated with program outcomes, and could be used to predict individually optimized settings. Such methods may significantly reduce the testing time required, while still providing significant improvement compared to the default settings for some individuals.

## Conclusion

When converting from Freedom to CP900 series sound processor, a significant improvement in speech understanding in quiet and in noise for the group mean was obtained. Individual analysis showed that 26 % of recipients received improved speech understanding in either quiet or noise with the default CP900 series sound processor settings. By selecting a program with individually optimized smart sound options, 39 % of the recipients showed a significantly improved speech understanding. To identify individually optimal settings, additional audiometric testing time would be increased by approximately 33 %.

The paper suggests that scene-dependent algorithm selections may further increase the overall speech intelligibility compared to default settings. If scene-dependent algorithm selections can be further individualized for a range of acoustic scenes, additional incremental increases might be expected, but would also involve extended audiometric testing.
